# Colchicine in Athero-Thrombosis: Molecular Mechanisms and Clinical Evidence

**DOI:** 10.3390/ijms24032483

**Published:** 2023-01-27

**Authors:** Giovanni Cimmino, Francesco S. Loffredo, Gennaro De Rosa, Plinio Cirillo

**Affiliations:** 1 Department of Translational Medical Sciences, Section of Cardiology, University of Campania Luigi Vanvitelli, 80131 Naples, Italy; 2 Department of Advanced Biomedical Sciences, Section of Cardiology, University of Naples “Federico II”, 80131 Naples, Italy

**Keywords:** colchicine, cardiovascular disease, inflammation

## Abstract

Several lines of evidence have clearly indicated that inflammation plays a pivotal role in the development of atherosclerosis and of its thrombotic complications such as acute coronary syndromes or ischemic stroke. Thus, it has been postulated that the use of anti-inflammatory agents might be extremely useful to improve cardiovascular outcome. Recently, increasing attention has been reserved to one of the oldest plant-derived drugs still in use in clinical practice, colchicine that has been used as drug to treat inflammatory diseases such gout or Mediterranean fever. To date, current guidelines of the European Society of Cardiology have included colchicine as first line choice for treatment of acute and recurrent pericarditis. Moreover, several studies have investigated its role in the clinical scenarios of cardiovascular disease including chronic and acute coronary syndromes with promising results. In this review, starting from a description of the mechanism(s) involved behind its anti-inflammatory effects, we give an overview on its potential effects in atherothrombosis and finally present an updated overview of clinical evidence on the role of this drug in cardiovascular disease.

## 1. Introduction

Current guidelines on cardiovascular disease prevention recommend low plasma levels of low-density lipoprotein cholesterol (LDL-C) to reduce the patient’s cardiovascular risk and atherosclerotic burden [1]. However, clinical and experimental data support an important and additional role of inflammation in the various steps of atherosclerosis, from its early stages till the acute final event represented by thrombosis after plaque rupture [2]. Indeed, many epidemiological studies have reported that some biomarkers of inflammation, such as high sensitivity protein C (hs-CRP) [3], interleukin-6 (IL-6) [4] and interleukin-1 beta (IL-1β) [5,6] are associated with the increased risk of developing cardiovascular events, regardless of cholesterol levels. Specifically, some studies have pointed out that CRP might play a role as prognostic marker [3], and others have indicated that the IL-6 and IL-1β have a causal role in the pathogenesis of atherosclerosis [4,5,6]. Indeed, it has been postulated that the benefic effects of statins observed in the context of cardiovascular prevention might be due not only to the lowering of cholesterol levels but also to the modulation of inflammation witnessed by the parallel reduction of plasma levels of some markers of inflammation [7,8]. Considering a pathophysiologic point of view, among the inflammatory mediators, IL-1β seems to be one of the main cytokine involved in the complex network of immune-inflammation linked to atherosclerosis. Interleukin-1 was suggested to be involved because it modulates the downstream pathway represented by IL-6-CRP axis [5,9]. More recent studies have shifted the point of view about relationship between CRP and coronary artery disease because they have clearly indicated that CRP might be no more considered not only a simple marker of disease but it is actively involved in acute coronary syndrome (ACS) pathophysiology. Indeed, several in vitro and in vivo studies have shown that CRP exerts direct biological effects on endothelial cells: (a) it promotes expression of adhesion molecules [10,11,12,13] and Tissue Factor (TF) [14]; (b) it stimulates release of monocyte chemoattractant protein-1 (MCP-1) [15] and matrix metalloproteinases (MMPs) [16,17]; (c) secretion of other inflammatory cytokines [18,19]; (d) increases inducible nitric oxide synthase (iNOS) and superoxide production and decreases endothelial nitric oxide synthase (eNOS), prostacyclin and tissue plasminogen activator (tPA) expression [11,20]. CRP sustains an anti-inflammatory innate immune response when circulates in a pentameric form, while as monomeric form, it exerts potent proinflammatory and prothrombotic actions. Interestingly, this conversion may occur at the site of plaque rupture, mainly induced by activated platelets [21,22]. In line with the pathophysiologic concepts reported above, in the Canakinumab Antiinflammatory Thrombosis Outcomes Study (CANTOS), canakinumab, a monoclonal antibody against IL-1β was administered on top of optimal therapy including statins in patients with high cardiovascular risk. Canakinumab administration was associated with a significant reduction of cardiovascular events in the absence of beneficial effects on blood pressure or cholesterol levels [23]. Interestingly, in this study, the extent of clinical benefit was proportional to the reduction plasma levels of hs-CRP or IL-6 achieved during treatment with the antibody [6,23]. The CANTOS represents the “proof-of-concept” that strongly outlined the importance of inflammation in atherosclerotic disease and demonstrated that the reduction of pro-inflammatory cytokine levels might be considered an additional target for the prevention of cardiovascular events. However, canakinumab therapy coincided with important side effects such as the increased risk of death due to infections and an unsatisfactory cost-effectiveness profile. Thus, more affordable anti-inflammatory strategies in the clinical scenario of cardiovascular disease should be considered. 

Newer concepts about atherothrombosis suggest that another pivotal mechanism involved in its pathophysiology is represented by immunity. Many evidences have outlined that an active immune response occurs in atherosclerosis contributing to plaque vulnerability [24,25,26,27,28,29]. Monocytes/macrophages are known to be involved in the initial stage of plaque formation [30], and other white blood cells, such as B and T-lymphocytes, are also involved in atherosclerosis [31,32]. Interestingly, despite of T-cells being a minority of the leukocytes enriching the plaques, several reports suggest their key regulatory role within atherosclerotic lesion [33] by modulating the adaptive immune response, by recruiting the more abundant monocytes/macrophages of the innate immune response [28,34]. Moreover, it has been clearly documented a specific, immune-driven response and inflammatory pathways within the “culprit lesion” of patients presenting with ACS [25], showing a specific oligoclonal T-cell expansion only in the vulnerable plaque and not in the peripheral blood indicating a selective expansion driven by a given intraplaque antigen. Taken together, these data strongly suggest that therapies against the immuno-inflammatory pathways involved in atherothrombosis might be extremely attractive to reduce the rate of acute coronary events. 

Recently, an increasing attention has been paid to an old and affordable drug, colchicine that has already used in clinical practice as anti-inflammatory drug, which had been recently proposed to treat cardiovascular disease too. This drug belongs to the tropolon family, and it is extracted from the plants of the genus Colchicum, in particular the Colchicum autumnale [35,36]. It is mentioned for the first time in the Ebers papyrus dating back to about 1500 years before Christ as an antirheumatic remedy of plant origin [36]. The drug has been used for centuries as an antirheumatic and antigout [35,37]. However, the active compound was isolated only in 1820 by two French chemists, PJ Pelletier and JB Caventon [36,38]. In 1833 PL Geiger purified the active ingredient and proposed the use of the term "colchicine" from Colchis, an ancient and legendary kingdom that overlooked the Black Sea and where Colchicum plants were very common with a typical autumn flowering [36,39]. To date, its use in cardiology is mainly for acute pericarditis and chronic pericardial disease [40]. Interestingly, some recent reports have indicated that colchicine possesses beneficial effects in patients with acute and chronic cardiovascular disease too [38,39]. However, the mechanisms by which colchicine exerts the observed cardiovascular effects have not completely defined yet. 

In this review the mechanism(s) involved behind its anti-inflammatory effects, its potential effects in atherothrombosis and finally an updated overview of clinical evidence on the role of colchicine in cardiovascular disease will be discussed.

## 2. Chemical Structure, Pharmacokinetics and Pharmacodynamics

N-{(7S)-5,6,7,9-tetrahydro-1,2,3,10-tetramethoxy-9-oxobenzo(a)heptalen-7-yl) acetamide} is the chemical structure of colchicine. It is a small lipophilic molecule composed of three rings (A, B and C) [41]. The rings A (trimethoxyphenyl moiety) and C (methoxytropone moiety) are mainly involved in binding to tubulin, while the ring B function is to maintain the structure in a rigid configuration [42]. Hence, modifications of A and/or C rings interfere with tubulin binding [43,44], while alterations on the B rings may modify activation energy of the binding and association/dissociation kinetics [45]. Colchicine is able to freely enter into the cells, from where it is actively removed via the glycoprotein -P (P-gp). The P-gp protein regulates colchicine absorption, distribution, and elimination. It is particularly expressed in the intestine, liver, kidney, central nervous system where it regulates the transit of substances and drugs [36]. Following oral route, colchicine bioavailability is extremely variable (ranging from 24 to 88% of the administered dose) and the mean apparent volume of distribution in young and healthy patients is calculated to be about 5–8 L/kg with moderate albumin binding. Its elimination occurred mainly via hepatic route with a half-life of 20 to 40 h [46]. At the dose of 1 mg/d, the steady state is reached 8 days after the first oral administration and plasma concentrations ranged from 0.3 to 2.5 ng/mL [46]. However, based on pharmacokinetic/pharmacodynamic studies, the biological effects of colchicine were not related to plasma concentrations but to intraleukocyte concentrations [35,46]. Drug interactions may occur when colchicine is associated to other drugs, which interact with cytochrome P450, and/or P-gp and modify renal and/or hepatic clearances. Common colchicine related side effects are the following: gastrointestinal upset, diarrhea, neutropenia, anemia, fatigue, and headache. Less commonly, colchicine can cause muscle pain, paresthesias, pale/gray appearance of skin or the tongue, or flu-like symptoms. The therapeutic drug monitoring of colchicine during these circumstances could allow to prevent the side effects [36,46].

## 3. Colchicine: Molecular Mechanisms and Effects on “Atherothrombotic” Cells 

Colchicine is able to interfere with cytoskeleton of cells because it binds specifically to tubulin, that is one of its main components [46]. This drug exerts a biphasic effect of microtubules as at low concentrations it stops the growth of microtubules, while at high concentrations it promotes their depolarization. These effects on cytoskeleton significantly impact cell biology because several physiologic functions of healthy cells such as mitosis and consequently replication, cytoplasmic vesicle trafficking and cell mobility are impaired [35]. Cytoskeleton and specifically microtubules are also essential for the correct positioning of selectins that are a group of transmembrane glycoproteins expressed by leukocytes, platelets and endothelial cells during inflammatory processes [47,48]. It is intuitive that colchicine, by altering the qualitative and quantitative expression of these proteins might be an attractive drug to modulate the atherothrombotic network involving endothelium, inflammatory cells and platelets [47,48]. Importantly, beyond this effect on microtubules, it has been reported that colchicine may interfere with the activation of the Nucleotide-Binding Domain, Leucine-Rich–Containing Family, Pyrin Domain–Containing-3 (NLRP3) inflammasome [49]. This inflammasome is one of the most important complexes which participates in the processes of pathogen clearance and tissue repair [50,51]. Upon activation, the inflammasome also promotes an inflammatory form of cell death named pyroptosis [51,52] and leads to the generation of active forms of inflammatory interleukins, first of all IL-1, also known as endogenous pyrogen [35]. In the pathophysiology of atherosclerosis, NLRP3 inflammasome seems to play a pivotal role since it has been shown that cholesterol crystals and oxidized low-density lipoproteins (oxLDLs) are actively involved in NLRP3 activation [53,54]. Several studies have pointed out the relationship between inflammasome and atherosclerosis in humans. For example, in the context of stable atherosclerotic disease it has been demonstrated that components of NLRP3 inflammasome are highly expressed in carotid atherosclerotic plaques [55] and that high levels of expression of NLRP3 correlate with the severity of coronary artery atherosclerosis [56]. Similarly, in the clinical scenario of ACS, the inflammasome has been shown to be primed in peripheral monocytes from ACS patients [57] and patients with ACS showed elevated levels of NLRP3, IL-1 and IL-18 compared to controls [58]. Thus, since several studies have confirmed that colchicine limits NLRP3 inflammasome activity, this drug might have a great potential role to limit atherosclerosis. Indeed, the mechanisms underlying its anti-inflammatory activity have not completely elucidated. Based on the available data, it has been suggested that colchicine inhibits the intracellular transport of ASC (the adaptor molecule apoptosis-associated speck-like protein containing a C-terminal caspase recruitment domain (CARD)) which prevents co-localization of NLRP3 components thus **limits** the consequent release of active IL-1β [59]. Moreover, it has been demonstrated that this drug causes reduction of protein expression of cleaved caspase-1 and IL-1β, without affecting mRNA levels of NLRP3 or IL-1β [57]. Again, other studies have shown that colchicine has stable interaction with the ATP binding pocket of the NAIP (neuronal apoptosis inhibitor protein), C2TA (MHC class 2 transcription activator), HET-E (incompatibility locus protein from Podospora anserina) and TP1 (telomerase-associated pro-tein) (NATCH) region of NLRP3 that is essential for its activation [60] and, finally, that it causes inhibition of pore formation [61], a key step in ATP-mediated NLRP3 inflammasome activation [50]. Taken together, all these mechanisms lead to limited NLRP3 inflammasome activity and IL-1β release in vitro and in vivo [62] and finally reduce inflammatory burden

A large mass of studies has significantly contributed to the mechanisms involved in the pathophysiology of atherotrombosis. Specifically, this complex scenario includes several actors such as vascular wall cells, endothelial and smooth muscle cells, immune cells, and cells directly involved in thrombosis such as platelets. Early stages of atherosclerosis are characterized by changes in biology of endothelial and smooth muscle cells that both are pathologically altered in a complex manner [63,64]. Colchicine seems able to modulate several biologic mechanisms of endothelial cells. At low doses it inhibits microtubule dynamics and cell migration, while at high concentration (up to 11.0 nmol/L) inhibits cell division. It has been reported that treatment of Familial Mediterranean fever (FMF) patients with colchicine reduced the level of asymmetric dimethlarginine (ADMA), thrombomodulin (TM), and osteoprotegerin (OPG), suggesting an endothelial protective effect of colchicine [65]. In the atherosclerotic plaque formation, modified lipoproteins, mainly oxLDLs, play a crucial role [66]. These lipoproteins bind to endothelial cells by a specific receptor named lectin-like oxidized low-density lipoprotein receptor-1 (LOX-1) [67], and are able to promote endothelial dysfunction since endothelial cells lose their anti athero-trombotic features. For example, it has been shown that endothelial cells stimulated with oxLDLs turn to a prothombotic phenotype since they express TF, the main activator of coagulation cascade, known to be involved in coronary thrombosis during ACS [68]. Experimental studies have suggested that the oxLDL effects on TF expression in endothelial cells might be prevented by colchicine [69]. Colchicine effect should be mainly related to well-known activity of the drug on microtubules and cytoskeleton [35,36]. It is known that in unstimulated endothelial cells, TF is stored in intracellular pools with a distinct perinuclear localization [70]. Upon cell activation, TF transolcates to cell surface where TF may be detected and become functional [71]. This phenomenon is part of the intracellular traffic of secreted and transmembrane proteins in vesicles, such as TF, that move along cytoskeletal tracks associated to the microtubule arrays [72]. By binding with high affinity to the specific domain of β-tubulin, colchicine induces depolymerization and inhibition of microtubule assembly, finally leading to cytoskeleton disarrangement [73] with interruption of TF transmigration from the intracellular pool to cell surface. Moreover, colchicine also modulates the NF-κB/IκB axis. It is known that the promoter for TF is under the direct control of this nuclear transcription factor that upon cell activation translocates to the nucleus inducing expression of several genes [74]. Several studies have clearly indicated that NF-κB inhibition reduces TF gene and protein expression [68,75]. Microtubules are involved also in this phenomenon and if they are destabilized by colchicine, NF-κB translocation is greatly reduced [76] and consequently, the pathway for TF biosintheys was not activated. The effect of oxLDLs on cells is mainly mediated by LOX-1. The oxLDL/LOX-1 complex activates several pro-inflammatory signaling pathways including NF-kB in vascular endothelial cells and macrophages [77], and even the synthesis of other LOX-1 molecules, thus inducing an amplificatory loop [78,79,80]. Expression of functional TF maybe also mediated by LOX-1 activation [81]. Since colchicine is able to reduce also LOX-1 expression [69], this might be another hypothetical additional mechanism of TF inhibition. 

Smooth muscle cells (SMCs) are another vascular cell population involved in the pathophysiology of atherosclerosis [82]. These cells actively participate in formation, progression and plaque stabilization via several ways: (a) migration to the inner layer and proliferation; (b) secretion of extracellular matrix proteins (such as collagen and proteoglycans) with the formation of fibrous caps at advanced stage; (c) intake of oxLDL and formation of SMC-derived foam cells; (d) recruitment of macrophages [67]. In this regard, colchicine has been reported to attenuate SMC proliferation and migration via inhibition of DNA synthesis and microtubule polymerization and depolymerization [83,84,85]. Additionally, colchicine decreased collagen secretory activity and decreased the cGMP level of SMCs [86]. It is still to be determined whether colchicine prevents intima-media thickness in patients with early stage of atherosclerosis and plaque rupture in advanced atherosclerotic lesions.

The role of immune-competent cells in athero-thrombosis is accepted and now well documented [87]. Since leukocytes, and in particular neutrophils, have a reduced number of P-gp molecules, the drug accumulates particularly in these cells, carrying out an anti-inflammatory action [88]. A majority of cellular effects depends on the colchicine concentrations [35]. It has been reported that at low concentrations (nanomolar) colchicine inhibits neutrophil chemotaxis and adhesion to the inflamed endothelium while at higher concentration (µM) it promotes shedding of L-selectin from neutrophils and prevents further recruitment [48,88]. Finally, it inhibits neutrophil release of IL-1, IL-8, and superoxide production [89]. Moreover, at therapeutic concentrations, colchicine decreased neutrophil-(NPA) and monocyte-platelet aggregation (MPA) [47]. Colchicine also inhibits monocite proliferation and differentiation in vitro [90]. More recently it has been reported that colchicine reduces vascular inflammation by dampening uptake of inflammatory leukocytes into plaques through altering the recruitment profile of circulating monocytes and neutrophils [91].

T-lymphocytes are major players in determinig the final fate of the plaque via several mechanisms such as cell-to-cell interaction, production and release of cytokines as well as regulation of macrophage activity [92,93]. T-cells have been found at the site of plaque rupture [93,94] and in coronary thrombi [95], thus indicating that T-cell activation might be another important mechanism in the pathophysiology of plaque vulnerability, both in humans [25,26] and in animal models [96]. Moreover, T-lymphocytes are also able to express functional TF, the main activator of the coagulation cascade, upon activation [81,95], thus actively participating to thrombus formation. A first study evaluating the effect of colchicine on human lymphocytes has been published in 1997 showing how colchicine is able to potentiate cyclic AMP levels in these cells in response to other agents that affect microtubule assembly [97]. A subsequent study, published in 1990, indicates how colchicine modulates the lymphocytes function in relation to fibroblast proliferation [98]. A report from our reserch group indicate another potential benefit in T-cells by inhibiting TF expression and activity also modulating NF-κB/IκB axis even on top of statin treatment [99]. 

Platelets activation is essential for the thrombotic manifestations upon atherosclerotic plaque complications [100]. A first report suggesting that colchicine might affect platelet aggregation has been published in 1980 [101]. The molecular mechanisms by which colchicine exerts this effect involve cytoskeleton rearrangement in platelets once activated specifically modulating the key proteins responsible for this process [102]. Cytoskeleton rearrangement results from the dynamics of myosin contraction and of actin-filaments that is mainly regulated by myosin phosphatase target subunit 1 (MYPT1) and Lin-11, Isl-1 and Mec-3 kinase-1 (LIM kinases) [103]. LIMK-1, in turn, exerts its activity on cofilin, a member of actin depolymerization factor (ADF)/cofilin (A/C) family that accelerates the off-rate from pointed end and the on-rate at the barbed end of the actin filament [104]. In most cell types, including platelets, cofilin activity is regulated by its phosphorylation/dephosphorylation on a highly conserved serine residue. Specifically, it is inactivated via phosphorylation by LIMK-1 and reactivated after dephosphorylation by slingshot protein phosphatases [105]. When cofilin is phosphorylated by LIMK-1, its affinity for actin is reduced while dephosphorylation activates the enzyme [104]. This balance permits rearrangement of cytoskeleton. Colchicine seems to inactivate MYPT/p-MYPT pathway as well as LIMK-1/p-LIMK and cofilin/p-cofilin pathways in platelets stimulated with adenosine diphosphate (ADP), collagen and thrombin activating receptor peptide (TRAP) [102]. In resting platelets, tubulin is appreciable as a typical marginal ring while in activatred cells it appears as a diffuse staining, indicating that platelet shape change involves cytoskeleton rearrangement. Conversly, in colchicine-treated platelets, the tubulin marginal band is replaced by microtubules depolymerization or fragmentation that appears as compacted “spots” impairing the cytoskeleton rearrangement [99] finally resulting in reduced platelet aggregation even in vivo in patients already on antiplatelet therapy [105]. Two reports in humans have shown that colchicine reduced β-thromboglobulin levels, a protein released during platelet activation, while no effect is observed on mean platelet volume that is a marker of platelet activity in FMF patients [106] and reduces leukocyte-platelet aggregation (both monocyte and neutrophil) as well as levels of surface markers of platelet activity, such as p-selectin and Procaspase Activating Compound-1 (PAC-1) (activated GP IIb/IIIa) when administered to healthy subjects [47]. It is reported that other possible effects on platelets might derive from that the anti-inflammatory action of colchicine on neutrophils. Activated neutrophils may expel their DNA and proteins forming an extracellular matrix, termed neutrophil extracellular traps (NETs) [107]. It has been shown that NETs can facilitate thrombosis by promoting platelet adhesion, activation, and aggregation, and also the accumulation of prothrombotic factors such as vonWillebrand factor and fibrinogen [108]. Since colchicine reduce NETosis in both patients with Behcet’s disease [109] or ACS [110], it might postulated a reduction on platelet activation/aggregation. 

**Figure 1:**
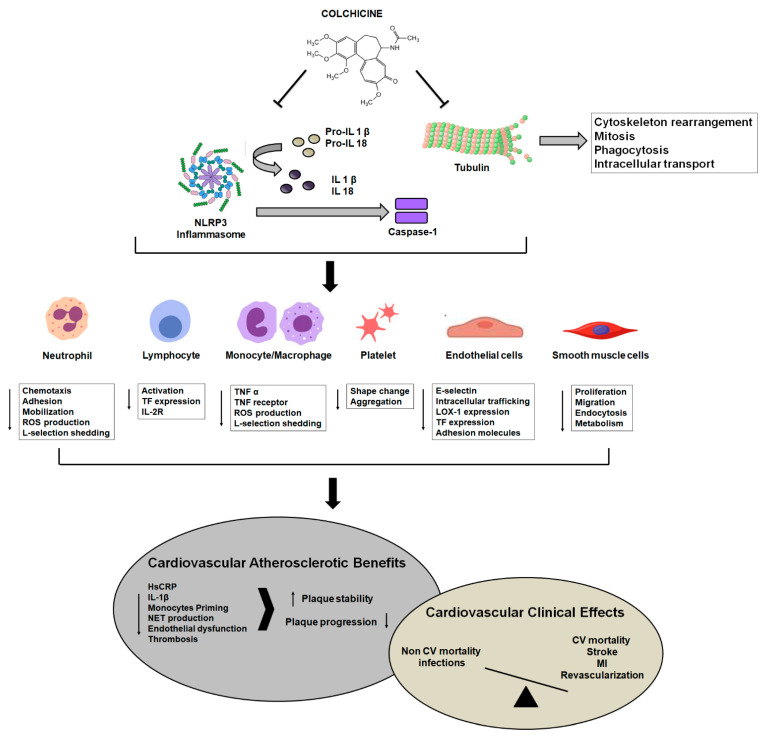
Effects of colchicine on key cells in atherothrombosis and potentials for cardiovascular benefits (with increased plaque stability and decreased plaque progression). TF, tissue factor; TNF, tumor necrosis factor; ROS, reactive oxygen species; LOX-1, Lectin-like oxidized low-density lipoprotein receptor-1; IL, interleukin; CV, cardiovascular; MI, myocardial infarction.

A schematic view of colchicine mechanisms on the key players in athero-thrombosis is reported in Figure 1.

## 4. Colchicine in Coronary Artery Disease: Clinical Studies

Considering the pathophysiologic as well as the experimental observations reported above, several studies have investigated the potential benefits derived by colchicine use in different cardiovascular scenarios such as chronic coronary artery disease, acute coronary syndromes and cerebrovascular disease. 

### 4.1. Chronic Coronary Syndrome 

The effects of colchicine in patients with coronary artery disease were initially investigated retrospectively in patients affected by diseases treated with this drug, such as FMF and gout. Specifically, patients with FMF in whom colchicine was prescribed long-term, had lower incidence of coronary disease as compared with not treated patients [111]. Similarly, other studies in which patients with gout treated with colchicine have shown a lower rate of coronary artery disease and of myocardial infarction [112,113]. Finally a larger retrospective study enrolling patients with gout also indicated that colchicine was associated with a signfiicat lower risk of the composite of myocardial infarction, stroke, transient ischemic attack and with reduction in all-cause mortality compared with patients not treated with this drug [114].

These restrospective studies were followed by two randomized controlled trials, the Low Dose Colchicine (LoDoCo) and the LoDoCo-2 trials. Specifically, the LoDoCo trial enrolled 532 patients with chronic coronary artery disease angiographically proven. This study showed that adding low dose (0.5 mg/day) colchicine to the optimal medical treatment significantly reduced the risk of acute coronary events, out-of-hospital cardiac arrests, and non cardioembolic ischemic strokes [42]. Thus, it was postulated that the observed effects were due to the ability of colchicine to inhibit the inflammatory responses that have been observed in unstable coronary plaques. The LoDoCo2 study was a larger study that included 5522 patients from Australia and Netherlands with chronic coronary artery disease treated with optimal medical therapy including statins [115]. In this study, colchicine led to a 31% reduction of cardiovascular death, ischemic stroke, myocardial infarction, and of ischemia-driven coronary revasculatization compared with placebo. Importantly, in these larger study the investigators did not observe any difference in hospitalizations due to infection, pneumonia or gastrointestinal disease between patients treated with colchciine and controls.

### 4.2. Acute Coronary Syndromes 

The effects of colchicine in patients with acute coronary syndromes were initially investigated in the Low Dose Colchicine after Myocardial Infarction (LoDoCo-MI) study [116], that evaluated the impact of low dose of colchicine on plasma levels of hs-CRP in 237 patients with a diagnosis of acute myocardial infarction. In this study the treatment with low dose of colchicine did not exert any effect on CRP [116]. Another study that investigated the effects of colchicine in the setting of acute coronary syndromes was the COVERT-MI multicenter study (Colchicine for Left Ventricular Infarct Size Reduction in Acute Myocardial Infarction) that enrolled 192 patients with a diagnosis of acute myocardial infarction treated with percutaneous coronary intervention (PCI) [117]. Patients were randomly assigned to 5 days of high-dose colchicine (2 mg dose followed by 0.5 mg twice daily) or placebo. This study failed in demonstrating benefits derived by colchicine treatment since no differences were demonstrated between groups in terms of infarct size, in biomarkers of inflammation, or in myocardial injury [117]. On the contrary, a higher number of patients with left ventricle thrombus in the colchicine group than in the placebo group at 5 days was observed, a difference that was no longer observed at 3 months [118]. A larger study, Colchicine in Patients With Acute Coronary Syndromes (COPS) trial, has investigated the effects of colchicine in the clinical scenario of ACS. A total of 795 patients admitted with a diagnosis of ACS (MI or unstable angina) were randomly assigned to receive colchicine (0.5 mg twice daily for one month, then 0.5 mg daily for other eleven months) vs placebo [119]. The original trial failed to demonstrate a significant difference between the two groups of patients in terms of all cause mortality, acute coronary syndromes, ischemia-driven urgent revascularization and non cardioembolic stroke, but a significant 40% reduction in the composite of all-cause mortality, ACS, ischemia driven-unplanned-urgent revascularization, and non-cardioembolic ischemic stroke were observed when follow up was extended till 24-month. 

The largest double-blind, placebo-controlled trial that evaluated the effects of colchicine in the clinical context of acute coronary syndromes was the COLchicine Cardiovascular Outcomes (COLCOT) trial [120]. In this trial, 4745 patients, enrolled within 30 days after acute myocardial infarction, were randomly assigned to receive 0.5 mg daily of colchicine on top of optimal mediacl therapy or placebo and were followed for 23 months. A 23% reduction of cardiovascular death, resuscitated cardiac arrest, MI, stroke, or urgent hospitalization for angina was observed in the colchicine treated patients [120]. These results were driven mainly by a significant reduction in the incidence of stroke and urgent hospitalization for angina leading to coronary revascularization. An interesting take-home message emerging by this study was that the greatest benefit in terms of clinical outcome was observed in patients treated with colchicine in the first three days after the acute event with a lack of benefit when the drug was started later. Another interesting result of the COLCOT study was that patients treated with low doses of colchicine had a markedly lower risk of stroke compared with the placebo group suggesting that this drug might exert benefic effects in this clinical scenario too [120]. 

A summary of the studies exploring the effects of colchicine in clinical practice is provided in Table 1.

### 4.3. Cerebrovascular Disease

To date, few clinical trials have specifically investigated the impact of colchicine therapy on cerebrovascular disease. However, several recent meta-analyses have clearly shown the impressive stroke reductions associated with colchicine compared **to** placebo. Of note, although these analysis included different studies and had controversial results on benefit derived by colchicine administration on cardiac endpoints, such as myocardial infarction and cardiac death, they were in line in demonstrating that colchicine had promising results in significantly reducing the risk of stroke [121,122,123,124]. To date, the effect of colchicine on non-cardioembolic strokes is evaluated by the ongoing CONVINCE (Colchicine for prevention of Vascular Inflammation in Non-CardioEmbolic Stroke) trial [124] that has planned recruitment of 2623 patients who have recently suffered a noncardioembolic transient ischemic attack or ischemic stroke [124]. 

## 5. Conclusions

The mechanisms by which colchicine exerts its cardioprotective effects might be multiple and still poorly understood. Fortunately, more recent data have contributed to explain the mechanisms by which this drug might exert its effects and they have shed a brighter light on the cardiovascular benefits of colchicine. In particular, grown attention in focusing on the anti-inflammatory effects of colchicine observed in cell types involved in atherothrombosis, such as endothelial cells, lymphocytes, and platelets. It might be hypothesized that the lower rate of future cardiovascular events in colchicine-treated patients might be due, at least in part, to the anti athero-thrombotic effects described in cells involved in the athero-thrombotic pathophysiology such as cells of vascular wall, of immunity and platelets. Further basic research and clinical studies should be done to reduce the gray areas that still persist about colchicine and its use in acute cardiovascular events. 

## Figures and Tables

**Table 1:** Clinical trials evaluating Colchicine effects in patient affectd by acute/chronic coronary syndrome.

STUDY	Target Population and Trial Characteristics	Primary Endpoint	Main Results	Ref.
LoDoCo trial 2013	Stable CAD	ACS	5.3% vs. 16%	[42]
	532 patients	Cardiac Arrest *	*p* < 0.001	
	Prospective, open label Colchicine 0.5 mg OD	Ischemic Stroke. ^#^ Median follow-up 3 years		
LoDoCo2 trial 2020	Stable CAD	CV death	6.8% vs. 9.6%	[116]
	5522 patients	MI ^§^	*p* < 0.001	
	Prospective, double-blind	Ischemic Stroke ^#^		
	Colchicine 0.5 mg OD	Revascularization °		
		Median follow-up 28.6 months		
COPS 2020	ACS	Total death	6.1% vs. 9.5%	[120]
	795 patients	ACS	*p* = 0.09	
	Prospective, double-blind	Revascularization ^£^		
	Colchicine 0.5 mg BID (1 month)	Ischemic Stroke ^#^		
	Colchicine 0.5 mg OD (11 months)	at 12 months		
COLCOT 2019	AMI within 30 days	CV death	5.5% vs. 7.1%	[121]
	4745 patients	Resuscitated Cardiac Arrest	*p* = 0.02	
	Prospective, double-blind	MI		
	Colchicine 0.5 mg OD	Stroke		
		Hospitalization ^&^ Median follow-up 22.6 months		

LoDoCo = Low Dose Colchicine; COPS = Colchicine in patients with acute coronary syndrome: the Australian Randomized clinical trial; COLCOT = Efficacy and safety of low-dose colchicine after myocardial infarction; * out of hospital cardiac arrest; ^#^ Non-cardioembolic ischemic stroke; ^§^ spontaneous non-procedural MI; ° ischemia-driven coronary revascularization; ^£^ ischemia-driven unplanned urgent revascularization; ^&^ urgent hospitalization for angina leadingto coronary revascularization; CAD, coronary artery diseases; AMI, acute myocardial infarction; CV, cardiovasacular.

## Data Availability

Data are available to corresponding author upon request.

## Abbreviations

ACS, acute coronary syndrome; ADP, Adenosine Diphosphate; AMP, Adenosine Monophosphate; ATP, adenosine triphosphate; CARD, C-terminal caspase recruitment domain; cGMP, cyclic guanosine monophosphate; COPS, Colchicine in Patients With Acute Coronary Syndromes; hs-CRP, high sensity C-reactive protein; DNA, Deoxyribonucleic Acid; eNOS, endothelial nitric oxide synthase; IL, interleukin; iNOS, inducible nitric oxide synthase; LIMK-1, Lin-11, Isl-1 and Mec-3 kinase-1; LOX-1, lectin-like oxidized low-density lipoprotein receptor-1; MCP-1, monocyte chemoattractant protein-1; MMP, matrix-metalloproteinases; mRNA, messenger ribonucleic acid; MYPT-1, myosin phosphatase target subunit 1; NACHT, NAIP (neuronal apoptosis inhibitor pro-tein), C2TA (MHC class 2 transcription activator), HET-E (incompatibility locus protein from Podospora anserina) and TP1 (telomerase-associated protein); NETs, neutrophil extracellular traps; NF-kB, nuclear factor kappa B; NLRP3, Nucleotide-Binding Domain, Leucine-Rich-Containing Family, Pyrin Domain-Containing-3; NPA, neutrophil-platelet aggregate; oxLDL, oxidized low-density lipoprotein; PAC-1, Procaspase Activating Compound-1; P-gp, P-glycoprotein; SMC, smooth muscle cells; TF, tissue factor; TNF, tumor necrosis factor; tPA, tissue plasminogen activator; TRAP, thrombin activating receptor peptide.
